# A case report of extra-adrenal retroperitoneal myelolipoma

**DOI:** 10.3389/fonc.2026.1788513

**Published:** 2026-03-06

**Authors:** Ying He, Junlin Li, Na Wei, Lan Zhang, Meiheng Liang

**Affiliations:** 1 Medical Imaging Department, Thirteenth People’s Hospital of Chongqing, Chongqing, China; 2 Department of Radiology, Chongqing Shapingba Maternity & Child Healthcare Hospital, Chongqing, China

**Keywords:** case report, computed tomography, extra-adrenal, myelolipoma, retroperitoneal

## Abstract

Myelolipoma is a benign tumor composed of mature adipose tissue and hematopoietic elements, typically found in the bone marrow. It mainly occurs in the adrenal gland, with only over 50 cases of extrarenal myelolipoma reported in literature, and extrarenal renal myelolipoma occurring in the posterior abdominal cavity is particularly rare. Currently, there are only 6 reported cases abroad. We report a case of extrarenal retroperitoneal medullary lipoma treated after abdominal pain, which was misdiagnosed as well differentiated retroperitoneal liposarcoma before surgery. The patient was successfully discharged after surgical resection. This paper provides a detailed summary of the clinical presentation, imaging features, diagnosis, and management course of this case, aiming to offer diagnostic and therapeutic insights for clinicians and future research.

## Introduction

Myelolipoma, also known as myeloadipose tumor, is a rare benign neoplasm that most commonly occurs in the adrenal glands, accounting for 6%–16% of adrenal incidentalomas (1). Extra-adrenal myelolipomas are extremely uncommon, with an incidence rate of about 0.08% to 2% (2), and may arise in locations such as the retroperitoneum, presacral region, mediastinum, and lungs (3). Patients usually have no obvious symptoms, but when the tumor is large, it can cause symptoms such as compression or bleeding (4).

Imaging examination is the main diagnostic method for myelolipoma, and computed tomography (CT) is the most commonly used modality to evaluate the lesion’s location, size, morphology, and relationship with adjacent organs. Magnetic resonance imaging (MRI) provides valuable supplementary information regarding the characteristics of the tumor’s composition. Research has shown that when medullary lipoma occurs in the retroperitoneum outside the adrenal gland, it is often misdiagnosed as retroperitoneal liposarcoma, causing overtreatment or unnecessary psychological burden on patients (5, 6). Accurately diagnosing the tumor before surgery undoubtedly poses a challenge for radiologists. In addition, because the global incidence rate of the disease is low, and the relevant imaging research evidence is still insufficient, our case can provide some imaging supplements for the disease.

Therefore, we will report in detail a case of preoperative diagnosis of liposarcoma and postoperative pathological confirmation of extrarenal retroperitoneal medullary lipoma, and retrospectively discuss and summarize its diagnostic points, aiming to improve clinical doctors’ understanding of this disease and achieve precise diagnosis and medical treatment.

## Case presentation

The patient is a 55-year-old male. He experienced intermittent, mild, dull pain around the umbilicus, which was tolerable and accompanied by radiating pain to the back two days ago. He had a history of hypertension, diabetes, and traumatic surgery. There is no family history of related tumors or genetic diseases.

**Figure 1:**
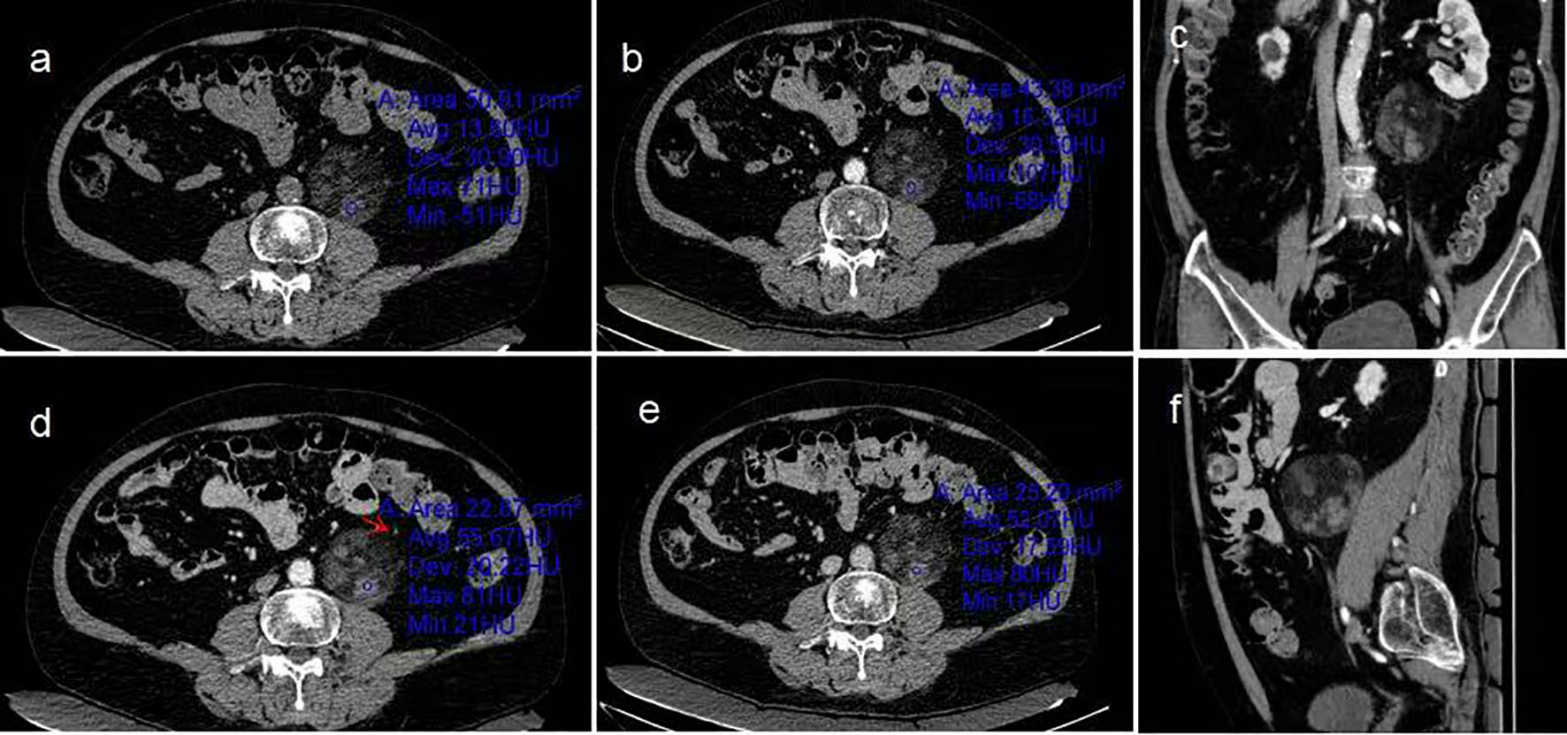
CT Non-contrast scan and enhancement. **(a)** Non-contrast abdominal CT scan shows a fat-containing heterogeneous density mass adjacent to the left side of the abdominal aorta in the retroperitoneum, **(b–f)** Contrast-enhanced abdominal CT scans demonstrate progressive moderate enhancement of some components within the mass, with no enhancement of the fatty components. The left ureter is compressed and displaced anterolaterally.

**Figure 2:**
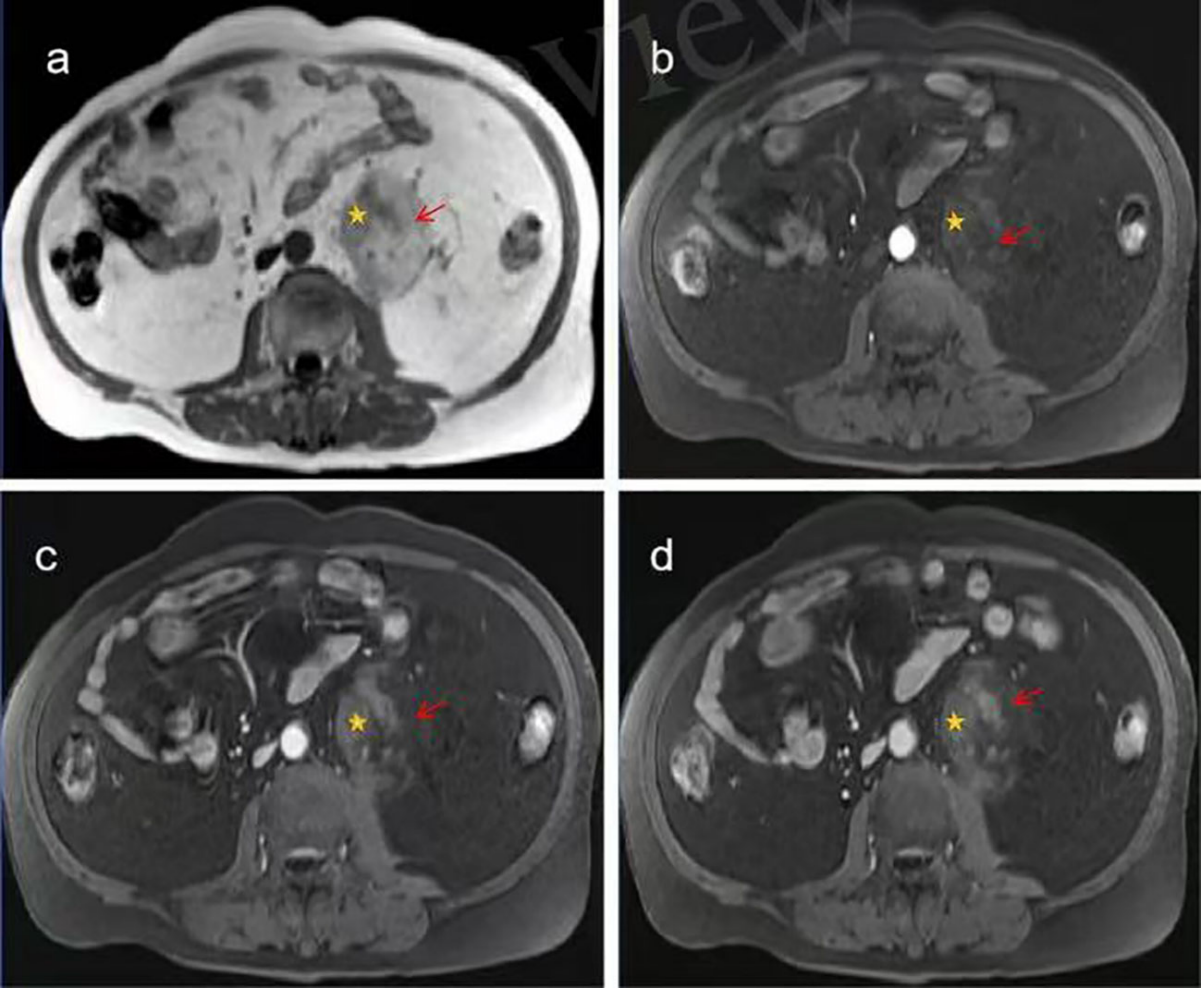
MRI images of extra-adrenal retroperitoneal myelolipoma. **(a)** Non-contrast abdominal MRI reveals a heterogeneous signal mass containing mature fat adjacent to the left side of the abdominal aorta in the retroperitoneum. **(b–d)** Contrast-enhanced abdominal MRI demonstrates progressive moderate enhancement in some components of the mass. with no enhancement of the fatty components (Mature adipose tissue, Lipid-containing myeloid tissue).

**Figure 3:**
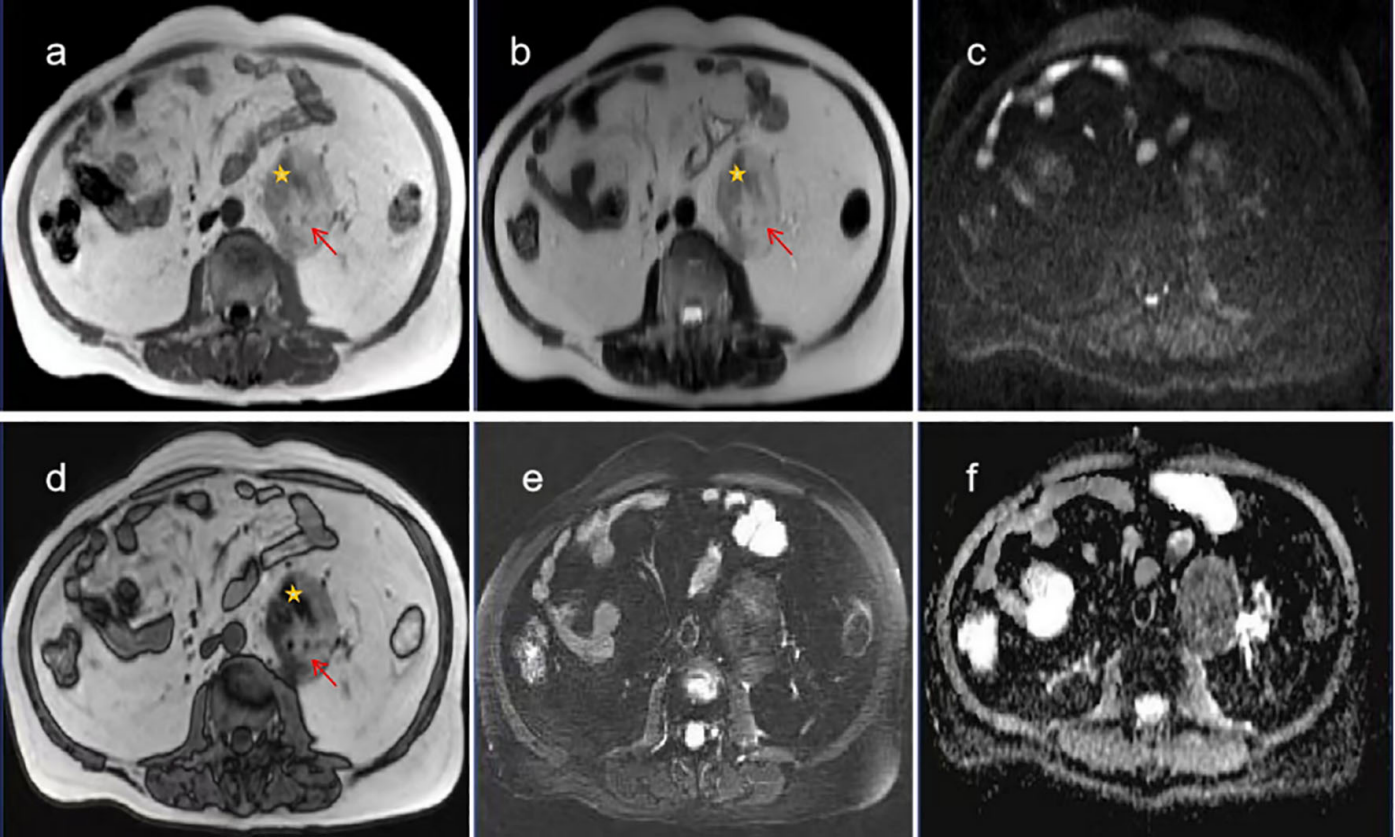
MRI images of extra-adrenal retroperitoneal myelolipoma. **(a–d)** Lipid components decrease in the reverse phase. **(b, e)** Mature fat decreases in the T2W1 lipid compression sequence. **(c, f)** DWI and ADC sequences show mild diffusion restriction in some components of the mass (Mature adipose tissue, Lipid-containing myeloid tissue).

After admission, the abdomen was found to be soft, and there was deep tenderness near the left side of the navel without obvious rebound pain. There are no special laboratory tests. No significant abnormalities were noted in the laboratory tests after admission. Computed tomography(CT) examination (**Figure 1a**) revealed a well-defined, heterogeneous mass measuring approximately 6.4 cm × 5.9 cm × 6.4 cm located lateral to the abdominal aorta in the retroperitoneum. The lesion demonstrated mixed density with CT values ranging from approximately -80 to 28 Hounsfield units (HU), predominantly composed of fatty tissue interspersed with scattered solid components. The left ureter was compressed and displaced anterolaterally. Contrast-enhanced CT scans (**Figures 1b–f**) showed mild enhancement of the solid components within the lesion and its capsule, while the fatty components exhibited no enhancement. Based on the CT findings, a neoplastic lesion was considered, with liposarcoma being the most likely possibility. Further enhanced magnetic resonance imaging(MRI) examination was recommended. MRI examination (**Figures 2a–f**) revealed a well-defined, heterogeneous mass approximately 6.7 cm × 5.8 cm × 6.4 cm in size located lateral to the abdominal aorta in the retroperitoneum. On T1-weighted imaging (T1WI) and T2-weighted imaging (T2WI), the lesion showed mixed high and slightly high signals. The high-signal components exhibited low signal on fat-suppressed T2-weighted sequences. The slightly high-signal components on in-phase T1-weighted imaging demonstrated signal loss on opposed-phase T1-weighted imaging. No obvious restricted diffusion was observed on diffusion-weighted imaging (DWI) or the apparent diffusion coefficient (ADC) map. The left ureter was compressed and displaced anterolaterally. Contrast-enhanced MRI (**Figures 3a–d**) showed mild enhancement of the soft-tissue components containing lipids and the lesion capsule, while the mature fatty components showed no enhancement. Preoperative MRI diagnosis: A space-occupying lesion lateral to the abdominal aorta in the retroperitoneum, suggestive of a neoplastic lesion, with well-differentiated liposarcoma considered most likely.

**Figure 4:**
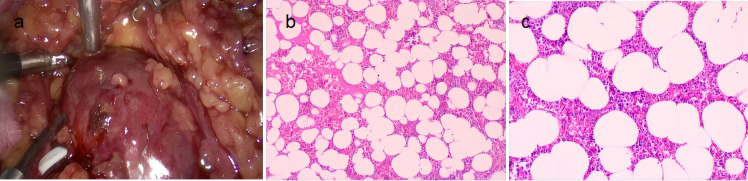
Pathological anatomy. **(a)** Intraoperative gross appearance of the tumor. **(b, c)** Observed under 100× and 200× magnification, cells of the erythroid, myeloid, and megakaryocytic series are diffusely distributed between adipocytes.

After discussing the patient’s condition, surgical resection was undertaken. Intraoperatively, a tumor measuring about 7.0 cm × 7.0 cm was identified in the left retroperitoneum. It had a complete capsule and showed adhesion to adjacent tissues but no invasion into blood vessels or the kidney. The mass had compressed the left ureter and was successfully removed in its entirety. Section examination (**Figures 4a–c**) revealed a collection of grayish-brown, fragmented soft tissue in the retroperitoneum, measuring 9.0 cm × 8.0 cm × 3.0 cm in total. The tissue was soft, resembling necrotic material, with small amounts of fatty-appearing tissue visible within. Pathological diagnosis confirmed myelolipoma. Immunohistochemical results showed (paraffin block) (delicate areas): CD34(-), Desmin(-), MDM2(-), CDK4(-), Ki-67(+, approximately 1%), S-100(+), Rb(intact), P53(-), P16(-), SMA(-), HMB45(-), Melan-A(-), MPO(+), CD71(+), CD61(+).

The patient undergoes regular dressing changes outside the hospital to prevent infection, and the patient’s compliance and tolerance are good. The patient reported improvement in abdominal pain symptoms during the 3-month follow-up after surgery, but did not undergo any relevant imaging examinations.

## Discussion

Myelolipoma is a nonfunctioning benign tumor capable of producing trilineage hematopoietic cells, including megakaryocytes, erythroblasts, and myeloblasts (7). It has been reported that the presence of megakaryocytes is considered a prerequisite for the diagnosis of myelolipoma (8). Myelolipomas can be classified into two types based on the proportion of their components: Type I is predominantly composed of adipose tissue, and Type II is mainly composed of myeloid tissue. Type I is more common than Type II (9). Our case, as confirmed by MRI, belongs to Type I. The pathogenesis of myelolipoma has not been fully elucidated. It has been proposed that there may be an association between elevated levels of erythropoietin or adrenocorticotropic hormone (ACTH) and the development of myelolipoma (10). Since the patient in this case exhibited no symptoms of anemia or Cushing’s disease preoperatively, relevant hormone tests were not conducted, making it impossible to determine the etiology. Myelolipoma occurs more frequently in females (male-to-female ratio approximately 1:2) and in middle-aged to older adults (50–70 years old), and is often discovered incidentally. Its size can range from 2 to 26 cm (2). Smaller tumors are typically asymptomatic. When the diameter exceeds 10 cm or the mass exceeds 1,500 g, it is classified as a giant myelolipoma with a risk of rupture and bleeding, which may be due to tumor enlargement and obvious capsule traction. Studies have shown that when the tumor is larger than 6cm, the risk of bleeding increases sevenfold (11). In addition, studies have shown a positive correlation between tumor size and increased risk of rupture and bleeding, with an overall rupture bleeding rate of 1.52 and all tumors larger than 7cm (12, 13). Additionally, larger tumors can compress adjacent tissues, leading to corresponding symptoms (14), with abdominal pain occurring in 47% of cases and gastrointestinal obstruction in 18% (15). The patient’s symptoms of periumbilical discomfort with radiation to the back are considered to be caused by compression of adjacent tissues due to the relatively large tumor size (7.0 cm × 7.0 cm as identified during surgery). Myelolipoma is a benign tumor with a low recurrence rate, and surgical resection is curative. For symptomatic or large tumors (≥5 cm), surgical excision is the treatment of choice; for asymptomatic, smaller tumors (<5 cm), dynamic imaging surveillance may be adopted (1, 7).

Myelolipoma lacks specific clinical manifestations and laboratory findings, and is primarily discovered incidentally during physical examinations. The typical CT appearance of myelolipoma is a heterogeneous density mass predominantly composed of fat, intermixed with streaky or patchy areas of slightly higher density. It usually presents with a complete or incomplete pseudocapsule, well-defined borders, and no signs of invasive growth. On contrast-enhanced scans, the myeloid tissue components show mild enhancement, while the fatty components exhibit no enhancement. The degree of enhancement correlates with the proportion of myeloid tissue (16). The imaging findings in this case are highly consistent with those reported in previous studies. In this case, hyperintense adipose tissue was observed on both T1-weighted imaging (T1WI) and T2-weighted imaging (T2WI) sequences, which exhibited signal loss on fat-suppressed T2-weighted sequences, indicating the presence of mature fatty tissue. The myeloid tissue appeared isointense on T1WI and slightly hyperintense on T2WI (17). Components showing slight hyperintensity on in-phase images demonstrated signal dropout on opposed-phase images, which differs from previous reports (18) and may be attributed to lipid-containing components within the myeloid tissue. Diffusion-weighted imaging (DWI) and apparent diffusion coefficient (ADC) sequences revealed no or only mild diffusion restriction within the tumor. Additionally, linear hypointensity on T1WI was noted at the lesion margin, suggesting the presence of a capsule.

Differential Diagnosis: The main differential diagnoses of myelolipoma are shown in **Table 1**. According to literature reports, technetium-99m sulfur colloid scintigraphy helps differentiate extra-adrenal myelolipoma from liposarcoma, as the former takes up the tracer while the latter typically does not (19). MRI signal characteristics of extramedullary hematopoiesis vary with hematopoietic cell activity. During the active phase, lesions show marked enhancement due to abundant vascularity, presenting with intermediate signal intensity on T1WI and high signal intensity on T2WI. In the remission phase, lesions predominantly contain hemosiderin deposition, showing low signal intensity on both T1WI and T2WI without significant enhancement. When fatty degeneration occurs, lesions exhibit high signal intensity on both T1WI and T2WI. Retroperitoneal angiomyolipoma can occur in multiple locations throughout the body. It consists of blood vessels, smooth muscle, and adipose tissue, with calcification being rare. Retroperitoneal lipoma is mainly composed of homogeneous adipose tissue, making diagnosis relatively straightforward. Despite these distinguishing features, similarities in presentation among these conditions may still lead to misdiagnosis. Therefore, a biopsy may be necessary to confirm the diagnosis and rule out potential malignancy.

**Table 1:** Differential diagnosis.

Disease name	Clinical manifestations	Imaging manifestations	Pathological features	Specific markers
Retroperitoneal liposarcoma	Abdominal pain, lumps, and strong invasiveness	Fat, soft tissue components, uneven reinforcement	Heteromorphicadipocytes, malignant	MDM2 Gene amplification(>90%)
Retroperitoneal vascular smooth muscle lipoma	Asymptomatic, prone to bleeding	Fat and vascularcomponents significantly enhance	A mixture of fat, blood vessels, and smooth muscle, benign	HMB-45、Melan-A Positive
Retroperitoneal extramedullary hematopoiesis	Have a history of blood disorders/splenectomy	Soft tissue adjacent to the spine, a small amount of fat, light reinforcement	Hematopoietic tissue hyperplasia, non-tumor	None
Retroperitoneal teratoma	More common among middle-aged and young people, often asymptomatic	Fat, cystic changes, calcification, or bone	Triple germ layer tissue, mature benign	AFP、β-HCG(Elevated in malignancy)
Retroperitoneal lipoma	Many asymptomatic, slowly increasing in size	Pure fat density, clear boundaries, no reinforcement	Mature adipocytes, benign	S100(Adipocytes)

## Conclusion

In summary, we described a rare case of symptomatic adrenal extra-adrenal retroperitoneal myelolipoma, which was successfully treated with surgical resection. Its imaging features are often indistinguishable from those of fat-containing retroperitoneal tumors. This case report reminds radiologists that when encountering retroperitoneal masses containing adipose tissue, adrenal extra-adrenal myelolipoma should be considered in the differential diagnosis. This approach can improve preoperative diagnosis and also assist clinicians in developing diagnostic and treatment strategies for such conditions.

## Data Availability

The original contributions presented in the study are included in the article/supplementary material. Further inquiries can be directed to the corresponding authors.
